# Fear of Fat, Processes of Change, and Weight-Related Behaviors in Mexican and Spanish Adolescents

**DOI:** 10.3390/children11080925

**Published:** 2024-07-30

**Authors:** María Marentes-Castillo, Isabel Castillo, Inés Tomás, Olivia González-Acevedo, Octavio Álvarez

**Affiliations:** 1 Department of Social Psychology, University of Valencia, 46010 Valencia, Spain; maria.marentes@ext.uv.es (M.M.-C.); isabel.castillo@uv.es (I.C.); 2 Department of Methodology of the Behavioral Sciences, University of Valencia, 46010 Valencia, Spain; ines.tomas@uv.es; 3 Institute of Nutrition and Food Technology “José Mataix Verdú”, Biomedical Research Center, University of Granada, 18017 Granada, Spain; oliviagonzalez@correo.ugr.es (O.G.-A.)

**Keywords:** fear of fat, processes of change, stages of change, weight-related behaviors, adolescents

## Abstract

Background: Fear of fat is a relevant variable in initiating weight regulation behaviors in adolescents. However, little is known about the experiential and behavioral change processes that mediate the choice between healthy and unhealthy weight regulation behaviors in this population. Method: The general objective was to examine the predictive role of fear of fat on weight-related behaviors (healthy and unhealthy weight control behaviors) through the mediation of experiential and behavioral change processes (consciousness raising, counterconditioning, helping relationships, reinforcement management, self-liberation, self-reevaluation, stimulus control, substance use) in 838 adolescents aged 15–19 years from Mexico and Spain using parallel mediation analysis. Additionally, the study examined the correlations between the variables with regard to gender and country, as well as the differences in the use of processes of change across stages of change through the use of a multivariate analysis of variance. The Spanish version of the Fear of Fat, Stages of Change, Processes of Change, and Weight-Related Behaviors scales were used. Results: The mediation analysis showed that fear of fat predicted healthy weight control behaviors through consciousness raising, self-liberation, and stimulus control. On the other hand, fear of fat predicted unhealthy weight control behaviors through counterconditioning, stimulus control, and the use of weight loss substances. Intervening to reduce fear of fat may be a way to modify the processes of change used by adolescents to regulate their weight. Conclusions: The results of this study indicate that adolescents may initiate weight control behaviors as a result of fear of fat. This process is mediated by experiential and behavioral processes that influence weight regulation.

## 1. Introduction

The fear of becoming overweight is a psychological phenomenon originated in the 1950s, a period during which the medical community established a direct association between the consumption of saturated fats and the risk of cardiovascular disease. This association led to the recommendation of low-carbohydrate and low-fat diets for weight loss and decreased cardiovascular risk [1]. At the same time, the emergence of a new perspective on health prevention as a form of health awareness movement, which has become known as ‘healthism’ [2,3], has contributed to the stigmatization of overweight people and obesity [4]. Consequently, the bias and prejudice towards being overweight can lead individuals to develop the “fear of fat,” which is conceptualized as the fearful attitude towards weight gain and being overweight [4,5]. Similarly, the fear of becoming overweight can be influenced by the pervasive media discourse surrounding the ideal of thinness, which can lead to dissatisfaction with one’s own appearance and current weight [6].

Adolescence represents a period marked by heightened vulnerability to risk and the emergence of maladaptive behaviors. These include poor dietary habits, a lack of physical activity, substance use, and risky sexual activity [7,8]. The adolescent population is notably preoccupied with body image and weight. They tend to hold the conviction that body weight is an essential factor influencing physical appearance, a perspective that has been empirically validated [9]. Platta et al. [10] observed that for young people, body weight is an important aspect of an attractive appearance. Body perception has an implicit relationship with well-being [11]. Weight control can be defined as a health-promoting behavior with the intention of reducing, maintaining, or preventing weight gain and achieving an optimal body image for each individual [12]. The regulation of weight is typically achieved through the combined influence of physical activity and a dietary plan that encompasses the consumption of fruits and vegetables, moderation in portion sizes, the reduction of processed foods and/or foods high in sugars and fats, and other similar dietary modifications [13,14].

Marentes et al. [15] found that fear of fat is a variable associated with the initiation of weight control in Mexican youths. Furthermore, youths who are in weight control have a greater fear of fat than those who are not in weight control. Dalley and Buunk [16] mentioned that weight loss strategies may be a first manifestation of the fear of fat. This is particularly evident in women who try to avoid the stigma of being overweight. Consequently, adolescents with a fear of fat may engage in unhealthy behaviors to control their weight, which may in turn lead to the development of eating disorder symptoms. These symptoms may contribute to the onset of other psychological conditions, suicidal ideation and behaviors, particularly in young women, as well as physical and psychological consequences and dissatisfaction with one’s body image [17,18]. Nevertheless, the variable fear of fat has not been extensively studied, although it has recently been identified as a phenomenon associated with unhealthy and weight control behaviors [4,19,20,21].

Kim et al. [22] demonstrated that adolescents with a normal weight are more likely to perceive themselves as overweight or obese than those who are obese, which leads them to engage in weight control behaviors more frequently. Marentes et al. [15] found that most Mexican youth employ both healthy and unhealthy behaviors when attempting to control their weight, regardless of whether they are in the action or maintenance stages. Furthermore, the pursuit of weight control is not limited to individuals who are overweight or obese. It also encompasses those who perceive themselves to be underweighted and who are attempting to avoid further weight gain [17,18,23].

To gain insight into adolescent weight control behaviors, numerous studies have been conducted on weight perception and body image, as well as associated factors and methods of control. However, few studies have explored how adolescents modify their behaviors or adopt healthy behaviors in terms of weight control, and the psychological strategies they employ to effect such change [9,24]. Therefore, the investigation of dietary patterns represents an essential component of any strategy designed to encourage individuals to adopt a healthier lifestyle. It is important to recognize that these dietary patterns often persist into adulthood [25].

In this context, the transtheoretical model of change has explained the process by which an individual transitions from a previous behavior to a new one and abandons a problematic behavior. The transtheoretical model of change posits two central constructs: the stages of change, which refers to the motivational disposition that indicates when people change (precontemplation, contemplation, preparation, action, and maintenance), and the processes of change, which are the covert and overt strategies that people use to adopt a change, indicating how people change [26,27]. The eight processes of change associated with weight control, as established by Rossi et al. [26], are as follows: consciousness raising, counterconditioning, helping relationships, reinforcement management, self-liberation, self-reassessment, stimulus control, and substance use. The processes of change can be understood as two first-order variables: experiential processes (consciousness raising, self-liberation, and self-reevaluation) and the behavioral processes of change (counterconditioning, helping relationships, reinforcement management, and stimulus control). Substance use can be considered both an experiential and behavioral process [26]. The use of these processes depends on the stage of change where the individual is. In general, the use of experiential processes of change tends to be used more frequently in the contemplation and preparation stages, while behavioral processes tend to be used in the action and maintenance stages. People in the precontemplation stage use processes of change the least [26]. The utility effect of stages of change and processes of change varies from moderate to large in studies of smoking, substance abuse, psychotherapy, exercise, and diet [27].

The process of “consciousness raising” involves problem awareness and feedback, which are used to break down individual barriers in order to change the problem behavior or adopt a new behavior. In the context of eating, “counterconditioning” (reciprocal inhibition) involves the substitution of a new behavior for the old problem behavior. In this case, the new behavior would be the adoption of a healthy one. “Helping relationships” involve the acceptance and support of others to change the problem behavior or adopt a new behavior. The term “reinforcement management” refers to the process of modifying the contingencies that control or maintain the behavior to be changed. This can be achieved by modifying the reinforcement of others and of the individual him/herself. This can be done by avoiding a healthy behavior and/or adopting a healthy behavior. The term “self-liberation” refers to the individual’s choice and commitment to change the problem. This includes the belief that the problem can be changed. The term “self-reevaluation” involves emotional and cognitive evaluation of the pros and cons associated with changing behavior. “Stimulus control” involves the elimination of cues or avoidance of situations that enhance the problematic behavior. “Substance use” refers to the use of drugs, alcohol, self-prescribed or physician-prescribed medications, diet aids, tobacco, caffeine, or other substances to control or lose weight [26,27].

Chae et al. [9] found that adolescents in the precontemplation stage who used self-reevaluation and stimulus control were less likely to progress to the contemplation stage. However, these same processes of self-reevaluation, stimulus control, and past experience increased the likelihood of transitioning to the action and maintenance stage. With regard to weight loss, it is suggested that those who are actively engaged in weight loss efforts will use a greater number of processes of change than those who are merely contemplating or preparing to lose weight, or those who are solely focused on maintaining their current weight [28].

Mexico tops the list of American countries with the highest prevalence (30%) of overweight and obesity in children and adolescents. Overweight and obesity in this population quadrupled from 4% to 18% worldwide between 1975 and 2016. Current data reveal that the prevalence of overweight in adolescents was 23.9% and obesity was 17.2% in women and men, increasing this prevalence by just over 5 percentage points between 2006 and 2022 [29].

A World Health Organization report [30] indicates that Spain has a higher prevalence of obesity than other European countries, including France, Germany, Italy, and Portugal. However, it is lower than in other countries, such as the United Kingdom, Greece, and Hungary. Despite an increase in the prevalence of obesity in Spain between 1987 and 2020, trends have stabilized in the last decade. This stabilization may be attributed, in part, to an increase in regular physical exercise from 22.4% in 2011 to 37.7% in 2022, and a concomitant decrease in sedentary leisure time behaviors, from 55.1% in 1993 to 36.4% in 2020 [31].

**Figure 1:**
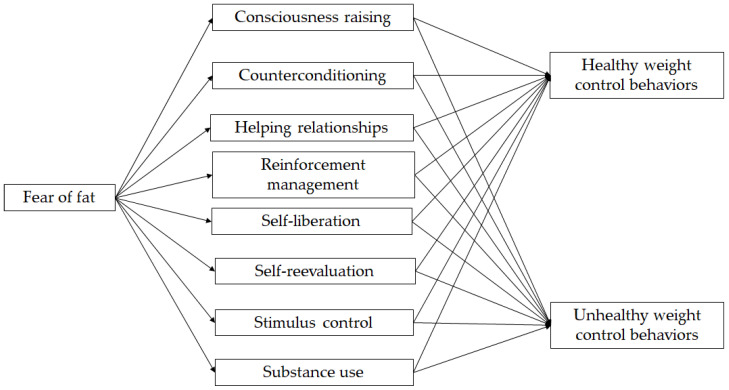
Parallel mediation model with multiple mediators between fear of fat and weight control behaviors. Note. The direction of the relationships between the variables was not established, as the processes of change do not have a theoretically established positive or negative valence.

According to extant literature and theoretical considerations regarding the potential role of fear of fat as a motivational factor in weight control behavior, the overarching objective of this study was to explore the predictive role of fear of fat on both healthy and unhealthy weight control behavior through processes of change within an adolescent population from Mexico and Spain (see Figure 1). This was achieved by controlling for country and gender, and by means of a parallel mediation analysis. Furthermore, the study examines the interrelationship between fear of weight gain, processes of change, and healthy and unhealthy weight control behaviors, with a particular focus on the influence of country and gender (first specific objective). Moreover, the study examines the discrepancies in the utilization of processes of change across stages of change (second specific objective). Two primary hypotheses are put forth. The first hypothesis posits that there will be a statistically significant correlation between fear of fat and healthy and unhealthy weight control behaviors. The second hypothesis suggests that processes of change may serve as potential mediators between fear of fat and healthy and unhealthy weight control behaviors.

## 2. Materials and Methods

### 2.1. Participants

A total of 838 adolescents from Mexico (*n* = 722) and Spain (*n* = 116) participated in the study. Of these, 579 were female (69.1%), 255 were male (30.4%), and 4 were of an unspecified gender. Of the respondents, 471 (56.2%) were in high school, 353 (42.1%) were in university, and the remainder (1.6%) were in secondary education. The participants were selected by non-probability cluster sampling from different schools in Mexico and Spain and ranged in age from 15 to 19 years (mean age = 17.50; standard deviation = 1.64). In order to be included in the study, participants were required to be enrolled in the academic year with regular attendance and aged between 15 and 19 years.

### 2.2. Instruments

The Goldfarb Fear of Fat Scale [5] translated into Spanish for this study, was used to assess the fear of gaining weight, differentiating between normal fear and out-of-normal fear. The scale contains 10 items (e.g., “My biggest fear is of becoming fat”) in a single factor, and employs a Likert-type response format, with options ranging from 1 (not at all true) to 4 (very true). The fit indices of the Confirmatory Factor Analysis (CFA) for this scale were found to be acceptable in this study (CFI = 0.97; TLI = 0.95; RMSEA = 0.08) (CFI = comparative fit index; TLI = Tucker–Lewis index; RMSEA = root mean square error of approximation. RMSEA values < 0.08, CFI and TLI values > 0.90 indicate a reasonable model fit).

The Stage of Change Questionnaire (URICA-Short form) [32] adapted to weight control and to Spanish [33] was employed. This scale categorizes individuals according to their stage of change (precontemplation, contemplation, preparation, action, and maintenance) with respect to weight control. The participants were asked to indicate whether they had attempted to lose or control their weight by selecting the option that best described their behavior: (1) Yes, I have done it for more than 6 months; (2) Yes, I have done it for less than 6 months; (3) No, but I will try in the next 30 days; (4) No, but I will try in the next 6 months; and (5) No, and I do not intend to try in the next 6 months.

The Processes of Change Questionnaire (PCQ) [26], translated into Spanish for this study, is a 32-item inventory designed to measure self-change processes used by individuals to cope with the problem of weight control. The inventory is composed of eight processes of change scales, each consisting of four items: (1) consciousness raising (“I weighed myself to keep track of my weight”), (2) counterconditioning (“When I was tempted to overeat, I thought about something else”), (3) helping relationships (“I could be open with at least one special person about my experiences with overeating behavior”), (4) reinforcement management (“I rewarded myself when I didn’t binge on food”), (5) self-liberation (“I told myself I could choose to overeat or not”), (6) self-reevaluation (“I thought about how upset I was when I binged on food”), (7) stimulus control (“I removed fattening foods from my home”) and (8) substance use (“I took diet aids to help me lose weight”). The responses were provided on a Likert scale, with options ranging from 1 (never) to 5 (repeatedly). In this study the fit for an 8-factor CFA model was adequate (CFI = 0.88; TLI = 0.87; RMSEA = 0.06).

The Spanish version [34] of the Weight-Related Behaviors Scale [35], was employed to assess both healthy and unhealthy weight loss behaviors. The scale comprises 15 items on a Likert-type scale ranging from 1 to 5 (never to always), divided into healthy weight control behaviors (6 items, e.g., “consume less sugar”) and unhealthy weight control behaviors (9 items, e.g., “consume very little food”). The fit indices for a 2-factor CFA model for this scale were acceptable (CFI = 0.94; TLI = 0.92; RMSEA = 0.07).

In accordance with the recommendations of the International Test Commission [36], the English versions of the Goldfarb Fear of Fat Scale and the Processes of Change Questionnaire, were translated into Spanish using the double translation and reconciliation procedure.

### 2.3. Procedure

This study was conducted in accordance with the ethical guidelines established by the American Psychological Association and with the guidelines established in the Declaration of Helsinki. All procedures involving participants in the research study were approved by the Experimental Research Ethics Committee of the University of Valencia (Ref: 1707311). The data collection took place from September to December 2022 for adolescents between the ages of 15 and 19 who were active in the school year. Contact was initiated with the Mexican and Spanish schools, and authorization was requested from their respective directors and coordinators to enable the collection of data from adolescents between the ages of 15 and 19. The survey was concluded with a total of 838 students from various educational institutions in Mexico and Spain. Data collection was conducted online using Google Forms. Prior to data collection, a live connection was established with the participants in order to explain the procedure and to ensure that the teacher in charge of the school group was present. Participants were made aware of their voluntary and anonymous participation in the study and were asked to complete an online form for approximately 15 to 20 min. This data collection in Mexican and Spanish adolescents represents a second phase of data collection within a larger research project. Several schools in Mexico and Spain were initially contacted, resulting in a discrepancy between the participants from both countries. This discrepancy can be attributed to the fact that Spanish students in the second data collection phase did not respond to the request for this data collection.

### 2.4. Data Analysis

Descriptive statistics provided information on the variables in this study. The percentage of missing data in the present study was less than 5% [37], indicating that it is unlikely to be a significant issue. To achieve the first specific objective, a correlational analysis was employed to determine the association between the variables under study in the total sample and also separately by gender. To compare the correlations across gender we used Fisher’s z-statistic. For the second specific objective of the study a multivariate analysis of variance (MANOVA) was conducted to determine whether the processes of change differed across the stages of change. In pursuit of the overall objective of this study, the association between fear of fat and the use of weight-related behaviors was tested, as well as the mediating role of the 8 processes of change, using Model 4 in SPSS macro-PROCESS (version 3.4.1.; IBM Corp., Armonk, NY, USA) with observed variables. Two separate parallel mediation models were run, one for each of the outcomes (healthy and unhealthy weight control behaviors), and both simultaneously, including the 8 processes of change as parallel mediators. The analysis included the examination of direct and indirect effects between variables and coefficients of determination (R^2^). The statistical significance was set at 0.05. The significance of the indirect effects was tested using 95% bootstrap confidence intervals, with 5000 replications [38]. The indirect effects were considered significant when the confidence interval did not include zero, thereby supporting a mediation effect.

## 3. Results

### 3.1. Descriptive Analysis

The descriptive data indicate that the sample, in general, presented a fear of fat below the mean. With respect to the change processes, all values were around or above the mean, except for substance use, which was below the mean. Moreover, the healthy weight control behaviors were slightly above the mean and the unhealthy weight control behaviors were below the mean. The normality values were in the acceptable ranges, except for substance use, which showed a kurtosis value outside the acceptable range. The alpha values were satisfactory for all the variables under study (see Table 1).

### 3.2. Correlational Analysis

The results of the correlations indicated that the fear of fat was positively and significantly related to consciousness raising, counterconditioning, reinforcement management, self-liberation, self-reevaluation, stimulus control, and substance use. Results highlighted the high value of correlation between the fear of fat and self-reevaluation. The fear of fat was not related with helping relationships. Furthermore, the fear of fat was positively and significantly related to healthy weight control behaviors and, in turn, to unhealthy weight control behaviors. Healthy weight control behaviors were positively and significantly related to all processes of change, and unhealthy weight control behaviors were also positively and significantly related to all processes of change, with the exception of helping relationships, which exhibit a very low, negative, and significant value (see Table 2).

A comparison of the correlation values between gender (see Table 3) revealed some statistically significant differences. The correlations of fear of fat with consciousness raising, self-reevaluation, and unhealthy weight control behaviors were all stronger for women than for men. Conversely, it is noteworthy that for the association between healthy weight control behaviors and helping relationships, self-liberation, self-reevaluation, and stimulus control, it was men who exhibit the highest values. Finally, women showed the highest values for the association between substance use and unhealthy weight control behaviors.

A comparison of the correlation values between countries (see Table 4) showed some statistically significant differences. The correlations of fear of fat with consciousness raising, self-reevaluation, reinforcement management, self-liberation, self-reevaluation, and substance use were all stronger for Spanish adolescents than for Mexicans adolescents. Also, for the association between healthy weight control behaviors and consciousness raising, counterconditioning, self-liberation, self-reevaluation, and stimulus control, it was Spanish adolescents who exhibited the highest values. Conversely, Mexican adolescents showed the highest values for the association between substance use and healthy weight control behaviors and unhealthy weight control behaviors. Finally, again Spanish adolescents showed a high and significant value for the relationship between helping relationships and unhealthy weight control behaviors.

### 3.3. MANOVA Analysis

As shown in Table 5, most participants were in the action stage, followed by the maintenance stage, the precontemplation stage, the preparation stage, and the contemplation stage. Males and females were not in all the stages of change at a similar percentage (x^2^(8) = 26.08, *p* = 0.001), with a low association between gender and stages of change for weight control (Cramer’s V = 0.13). Concretely, according to the adjusted standardized residuals, there were statistically significant differences in the precontemplation stage, preparation stage, and maintenance stage, with more male than female in the first two stages, and with more female than male in the last one.

**Figure 2:**
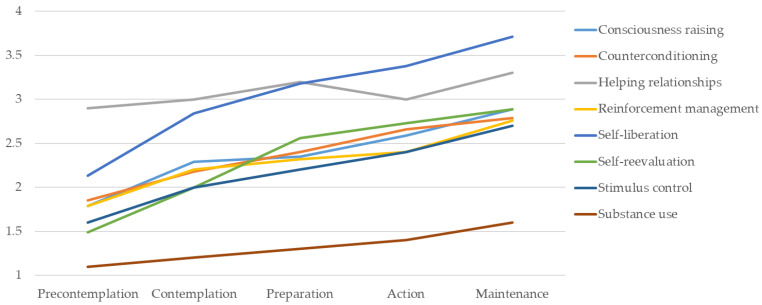
Use of processes of change across stages of change.

All processes of change seemed to exhibit a pattern of increasing use through the stages of change (see Table 6 and Figure 2). Participants in the precontemplation stage showed the lowest use of the processes of change compared to the participants in other stages. The use of consciousness raising did not differ significantly between participants in the contemplation, preparation, and action stages. However, there was a significant increment in its use for the participants in the maintenance stage in comparison with the participants in the precontemplation stage. In the case of counterconditioning, it should be noted that its use did not differ significantly between participants in the precontemplation and contemplation stages, between participants in the preparation and action stages, and between participants in the action and maintenance stages. However, there was a significant increment in its use between the participants in the maintenance stage when compared with participants in the precontemplation stage, as well as between participants in the action and contemplation stages, and between participants in the preparation and precontemplation stages. Helping relationships showed a higher use in participants in the maintenance stage when compared to participants in the precontemplation stages, but their use was similar for participants in the contemplation, preparation, and action stages. Statistically significant differences were observed between participants in the precontemplation and maintenance stages in terms of reinforcement management. However, no differences were evident in their use between participants in the contemplation, preparation, and action stages. Nevertheless, differences were observed between participants in the contemplation, preparation, and action stages when compared to participants in the maintenance stage.

The self-liberation strategy showed a higher use for the participants in the maintenance stage when compared to participants in the precontemplation stage. However, its use did not exhibit a significant divergence between participants in the contemplation and preparation stages, and between participants in the preparation stage and the action stage. With regard to self-reevaluation, its use was found to be higher within the participants in the maintenance stage when compared to the participants in the preparation stage, as well as between the participants in the contemplation stage when compared to participants in the precontemplation stage. Nevertheless, its use remained consistent between the participants in the preparation stage, the action stage, and the maintenance stage. In the use of stimulus control, there were no statistically significant differences between the participants in the precontemplation stage and the contemplation stage, nor between the contemplation stage and the preparation stage. However, there was a significant increase in the use of stimulus control for participants in the action and maintenance stages. Furthermore, both of these stages differ from the previous stages. With regard to the use of substances, no statistically significant differences were observed between participants in the first three stages (precontemplation, contemplation, and preparation). However, statistically significant differences were identified between participants in the precontemplation and action stages, with the last ones showing higher use. Furthermore, the use of substances for participants in the maintenance stage was found to be significantly higher when compared with participants from all the other stages of change.

### 3.4. Mediation Analysis

**Figure 3:**
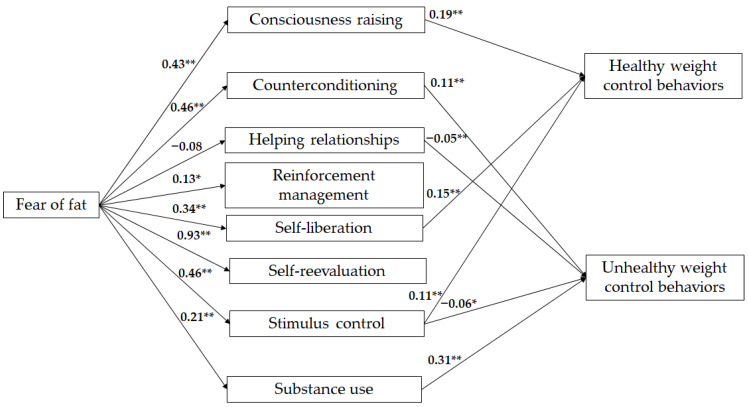
Unstandardized solution of the parallel mediation model with multiple mediators between fear of fat and weight control behaviors. For clarity, only significant relationships were placed in the second part of the model. * *p* < 0.05; ** *p* < 0.01.

The proposed model included gender and country variables as control variables to account for significant gender and country differences. To assess the proposed model (see Figure 1), we conducted two parallel mediation analyses with multiple mediators (one for each set of outcome variables). We tested whether processes of change mediate the effect of fear of fat on healthy and unhealthy weight control behaviors. Figure 3 and Table 7 show the results of the relationships between fear of fat and the processes of change, as well as between the processes of change and healthy and unhealthy weight control behaviors.

The fear of fat was found to be a significant and positive predictor of the following processes: consciousness raising, counterconditioning, reinforcement management, self-liberation, self-reevaluation, stimulus control, and substance use. Healthy weight control behaviors were positively predicted by consciousness raising, self-liberation, and stimulus control. Unhealthy weight control behaviors were positively predicted by counterconditioning and substance use and negatively predicted by helping relationships and stimulus control. The results indicated that the variables in the models explained the following percentages of variance: 20% for healthy weight control behaviors and 40% for unhealthy weight control behaviors. These percentages were statistically significant (see Table 7).

Table 8 shows that the relationship between fear of fat and healthy weight control behaviors was significant through consciousness raising, self-liberation, and stimulus control. Conversely, the relationship between fear of fat and unhealthy weight control behaviors was significant through counterconditioning, stimulus control, and substance use.

## 4. Discussion

The objective of this study was to examine the role of fear of fat as a predictor of healthy and unhealthy weight control behaviors through the processes of change. Fear of fat has been a relatively understudied variable. However, recent evidence indicates that it is a significant factor with a negative connotation that can influence the onset and control of weight [4,15,20,21]. Additionally, the study examined the correlations between the variables and the differences through gender and country, and finally, the differences in the use of processes of change across stages of change.

As a first specific objective, we analyzed the positive and significant correlation values between the fear of fat and the processes of change. This analysis indicated that the greater the fear of fat, the more frequently processes such as consciousness raising, counterconditioning, reinforcement management, self-liberation, self-reevaluation, stimulus control, and substance use are used. We emphasized the high correlation value between fear of fat and self-reevaluation, indicating the profound emotional involvement of fear of fat in young people. This emotional involvement could potentially lead to a range of adverse consequences, including physical and psychological distress, as well as dissatisfaction with one’s body image, as previously documented in numerous studies [17,18].

Conversely, the fear of fat is positively associated with both healthy and unhealthy weight control behaviors. These results provide valuable insight into the negative impact of fear of fat on the performance of negative weight control behaviors, as well as the initiation of healthier behaviors. Previous investigations [17,18] have highlighted that adolescents with a fear of fat may engage in unhealthy weight control behaviors. Marentes et al. [15] have additionally emphasized that fear of fat is a variable associated with the initiation of weight control in Mexican youth, with those who are controlling their weight exhibiting a greater fear of fat than those who are not.

Similarly, in this study, it was found that processes of change are positively associated with both healthy and unhealthy weight control behaviors. However, the relationship was found to be negative in the case of unhealthy weight control behaviors and helping relationships. This negative correlation indicates that as one does not seek support or rely on others for change, one is more likely to engage in unhealthy behaviors in order to change or vice versa. These results are noteworthy because they indicate that regardless of the valence of weight control behaviors, they will be associated with the use of the processes of change. In this sense, Marentes et al. [15] highlighted that most Mexican youths engage in weight control using both healthy and unhealthy behaviors when they are in the action and maintenance stages, that is, when they are carrying out weight control.

A more detailed examination of the correlations obtained reveals some interesting differences between males and females. Despite few differences between males and females, it is noteworthy that women exhibit higher levels of association with regards to fear of fat and consciousness raising, self-reevaluation, and the use of healthy weight control behaviors. This suggests that women are more likely to view weight as a problem, engage in constant self-feedback, and associate the fear of fat with a lack of health rather than a health issue. Nevertheless, it can be observed that the implementation of change or the constant regulation is associated with a greater use of healthy weight control behaviors in women. This indicates that youth women who undergo such changes are more likely to choose healthy behaviors to control or lose weight, despite the fact that the initial catalyst for change is not a change in health status, but rather the fear of fat.

Conversely, men exhibited the highest values in the associations between helping relationships, self-liberation, self-reevaluation, and stimulus control with healthy weight control behaviors. Finally, women exhibited a higher correlation value between substance use (e.g., excessive caffeine consumption, laxatives, medications, anorexigenics, thermoregulators) and unhealthy weight control behaviors, such as restrictive diets, prolonged fasting, use/abuse of laxatives, among others [39]. Over the past three decades, there has been a notable increase in concerns related to body weight and body image in the female population, as well as in the prevalence of risk behaviors [40]. This may be further compounded by social stigmas towards women, including those perpetuated through social media platforms, such as app filters, among others. A study conducted with Mexican female adolescents (aged 14 to 18 years) found that the internalization of the aesthetic model of thinness increases the risk of developing risky eating behaviors and attitudes by a factor of 11.8 [41].

In the relationships identified among Mexican and Spanish adolescents, it is noteworthy that the highest and most significant values observed in Mexicans are in the use of substances for both types of weight control behaviors (healthy and unhealthy), indicating that there will be a greater tendency to use external products to initiate or carry out weight control. In contrast, Spanish adolescents exhibit higher values in the fear of fat and change processes, suggesting that as they experience heightened concerns about weight gain, they are more likely to engage in change processes to achieve healthier weight management. It is also important to note that, as Spanish adolescents do not utilize helping relationships, there will be a greater reliance on unhealthy behaviors to control weight. The findings of the present study suggest that Spanish youth may demonstrate a more diverse utilization of change processes compared to Mexican youth, who may exhibit a greater proclivity to employ external substances and products to regulate weight.

With regard to our second specific objective, the discrepancies in the use of the processes of change and the stages of change was tested. It was observed that the application of processes did not vary considerably across participants in different stages. However, their use did tend to increase gradually as the participants were in more advanced stages. As previously noted by Rossi et al. [26], we concur that processes of change are utilized less frequently during the precontemplation stage, which is to be expected given that individuals in this stage are typically unaware of the problem or lack motivation to change [27]. Additionally, it is noteworthy that helping relationships appear to play a pivotal role in the maintenance stage, in contrast to the preceding stages where other experiential and behavioral processes may be more influential.

The preceding obtained results prompted us to run our general objective, the mediation model for the overall sample. However, we proceeded to control for gender and country within the mediation analysis. The results indicated that indeed, processes of change mediate the association of fear of fat and healthy weight control behaviors and unhealthy weight control behaviors. The results also supported that fear of fat has a predictive role in the initiation and maintenance of healthy and unhealthy weight control behaviors, with a higher percentage of variance over unhealthy weight control behaviors. This indicates that fear of fat may predict the use of unhealthy weight control behaviors in young adolescents, but that this is mediated by processes of change. Nevertheless, not all processes serve as a mediator between fear of fat and healthy and unhealthy weight control behaviors. In the case of healthy weight control behaviors, only consciousness raising, self-liberation, and stimulus control played a mediator role. This indicates that awareness of the problem, receiving and giving feedback on the problem, the choice and commitment to change made by young people, and the control of external signals that exist in the context of young people (calorie-dense foods, alcohol, sugary drinks, and processed and ultra-processed foods) are elements that are both experiential and behavioral processes that mediate the relationship between fear of fat, and the use of healthy weight control behaviors, thus, can increase the likelihood that youth will increase their physical activity levels and water consumption, decrease sugar and fat consumption, incorporate fresh foods such as fruits and vegetables, and distribute and balance total food consumption.

Conversely, the fear of fat may explain the occurrence of unhealthy weight control behaviors through the mediation of counterconditioning, stimulus control, and substance use (behavioral processes). These results indicate that counterconditioning and substance use may increase the probability of using unhealthy weight control behaviors. These behaviors include behavioral strategies such as behavioral substitution in eating behavior, which involves changing an unhealthy food for a healthy one (e.g., substituting sugary drinks for water). Another example is excessive caffeine consumption, and the use of laxatives in different presentations may also increase the probability of using unhealthy behaviors to control or lose weight. In contrast, the use of stimulus control, which involves regulating the availability of unhealthy foods and beverages (e.g., controlling the presence of calorie-dense foods, alcohol, sugary drinks, and processed and ultra-processed foods), can serve as a protective factor against the use of unhealthy behaviors to control or lose weight. Although no similar studies have tested these relationships, Chae et al. [9] found that change processes, such as self-reevaluation and the use of stimulus control, could cause different effects depending on the stage at which they are used. For instance, if stimulus control and self-reevaluation were used in the precontemplation stage, there was less likelihood of moving to the contemplation stage. However, if these same processes of change were used in the preparation stage, there was a greater probability of moving more quickly to the action and maintenance stages. In the case of the results of this study, it can be indicated that stimulus control can be a process of change that, if used in the family context, can lead young people to control or lose weight in a healthy way. In the case of counterconditioning, it may be recommended that the substitution of unhealthy foods for healthy ones be implemented through a successive approach, whereby healthy foods are introduced, and unhealthy products are decreased.

The aforementioned results corroborate the initial hypotheses, which postulate a statistically significant correlation between fear of fat and both healthy and unhealthy weight control behaviors. Furthermore, the processes of change serve as mediators in the relationship between fear of fat and healthy and unhealthy weight control behaviors among adolescents.

This study serves to confirm that the fear of fat can be considered a variable that can explain the initiation and maintenance of weight control. It thus demonstrates the importance of intervening from the juvenile fear of fat. This study represents a significant advancement in our understanding of the mediating role of the processes of change in the effect of fear of fat. It allows us to observe how certain processes of change are mediating mechanisms in the relationship between fear of fat and the use of healthy weight control behaviors, while also recognizing that their use can be associated with unhealthy behaviors (e.g., for counterconditioning and substance use). This leads us to suggest that we must focus and direct the beneficial use of the processes of change for their effectiveness through healthy nutritional strategies that incorporate the whole family and where complete and balanced nutrition prevails. Furthermore, we must apply cost-effective strategies using locally sourced and affordable food. Additionally, it is crucial to facilitate communication within the family and in the social context surrounding young people, enabling them to engage in dialogue and express concerns about weight gain, challenge prejudices and stigmas associated with individuals with higher weight, and gain a deeper understanding of the physical and physiological processes that are part of the adolescent stage and are related to weight gain. It is crucial to implement comprehensive nutritional and psychological education programs to address the factors associated with weight gain and/or weight control, with a particular focus on families and educational contexts.

The limitations of the study include the disparity between the Mexican and Spanish samples, even though country was introduced as a control variable to consider those potential differences. Other limitations include the fact that the variables were evaluated in a self-referenced manner and that no anthropometric measurements were taken of the sample under study. We are aware that self-reported data can be subject to biases, such as social desirability and recall errors, which may impact the accuracy of the reported weight-related behaviors and attitudes. Thus, the absence of anthropometric measurements precludes verifying the actual weight status of the participants, which might affect the interpretation of how fear of fat and processes of change relate to actual weight-related behaviors. Future studies should aim to include objective measures to enhance the robustness of the findings. Furthermore, the study employed a cross-sectional approach that precludes establishing any causal relationships between fear of fat, processes of change, and weight-related behaviors. The associations observed in this study are correlational, and thus, we cannot determine the directionality or causality of these relationships. Longitudinal studies are necessary to examine the temporal sequence of these variables and to draw more definitive conclusions about causality. Future research should employ longitudinal or experimental designs to better understand the causal pathways involved.

Future proposals could include identifying the perception that young people have of their own weight and body image in order to ascertain whether these two variables can differentiate the use of the processes of change, and also, to deepen which processes of change (experiential and behavioral processes) are most significantly used by different stages of change, as well as to document Rossi et al. [26]. Furthermore, objective measurements of weight and anthropometry should be included, as well as a qualitative deepening of the experiential and behavioral processes associated with the fear of fat. Additionally, it is necessary to evaluate the role of helping relationships in young people, although their role was not so clear in this study. It seems to be particularly relevant in the maintenance stage, which could also indicate the difficulty of young people to ask for help and to trust others when it comes to losing or controlling weight.

## 5. Conclusions and Practical Implications

The findings of this study indicate that young people may initiate weight control motivated by the fear of fat. This fear may be followed by processes of change, such as consciousness raising, self-liberation, and stimulus control in the case of healthy behaviors, as well as processes such as counterconditioning and the use of substances that increase the probability of unhealthy behaviors and stimulus control, which may decrease the probability of using unhealthy behaviors.

Overall, this study represents a significant advancement in understanding how adolescents change and adopt healthy behaviors, specifically weight control, through eating behaviors. The knowledge and preparation that nutrition professionals and families can obtain about the change processes that can be used for effective behavior change will be crucial for a healthy approach to dietary change, strategies to control weight, and reducing the fear of gaining weight.

## Figures and Tables

**Table 1:** Descriptive statistics, and reliability of the study variables (*n* = 838).

Variables	Range	Mean	SD	Skewness	Kurtosis	Alpha
Fear of fat	1–4	1.85	0.73	0.98	0.22	0.91
Consciousness raising	1–5	2.56	0.80	0.22	−0.25	0.70
Counterconditioning	1–5	2.56	0.93	0.18	−0.66	0.73
Helping relationships	1–5	3.11	1.17	−0.13	−0.99	0.85
Reinforcement management	1–5	2.44	0.95	0.45	−0.38	0.75
Self-liberation	1–5	3.30	1.08	−0.37	−0.58	0.84
Self-reevaluation	1–5	2.59	1.12	0.39	−0.76	0.83
Stimulus control	1–5	2.39	0.94	0.33	−0.59	0.72
Substance use	1–5	1.40	0.71	2.26	5.13	0.80
Healthy weight control behaviors	1–5	3.29	0.82	−0.03	−0.16	0.73
Unhealthy weight control behaviors	1–5	1.61	0.60	1.56	2.82	0.72

Note: SD = standard deviation.

**Table 2:** Bivariate correlations of the study variables (*n* = 838).

Variables	1	2	3	4	5	6	7	8	9	10
1. Fear of fat										
2. Consciousness raising	0.41 **									
3. Counterconditioning	0.36 **	0.41 **								
4. Helping relationships	−0.05	0.17 **	0.18 **							
5. Reinforcement management	0.19 **	0.38 **	0.40 **	0.50 **						
6. Self-liberation	0.25 **	0.43 **	0.45 **	0.41 **	0.57 **					
7. Self-reevaluation	0.62 **	0.50 **	0.50 **	0.17 **	0.36 **	0.50 **				
8. Stimulus control	0.37 **	0.49 **	0.48 **	0.26 **	0.50 **	0.51 **	0.60 **			
9. Substance use	0.22 **	0.29 **	0.20 **	0.07	0.30 **	0.15 **	0.22 **	0.33 **		
10. Healthy WC behaviors	0.16 **	0.34 **	0.27 **	0.13 **	0.29 **	0.35 **	0.25 **	0.32 **	0.11 **	
11. Unhealthy WC behaviors	0.49 **	0.28 **	0.32 **	−0.09 **	0.12 **	0.13 **	0.36 **	0.24 **	0.45 **	0.17 **

Note: WC = weight control. ** *p* < 0.01.

**Table 3:** Results of values of correlation differences by gender for the study variables.

Variables Correlated	Male (*n* = 255) Correlation	Female (*n* = 579) Correlation	Fisher z Coefficient
Fear of fat—Consciousness raising	0.29 **	0.44 **	2.23 *
Fear of fat—Counterconditioning	0.33 **	0.37 **	0.52
Fear of fat—Helping relationships	0.04	−0.09 *	−1.77
Fear of fat—Reinforcement management	0.20 **	0.07	−1.76
Fear of fat—Self-liberation	0.31 **	0.22 **	−1.35
Fear of fat—Self-reevaluation	0.54 **	0.65 **	2.26 *
Fear of fat—Stimulus control	0.40 **	0.35 **	−0.84
Fear of fat—Substance abuse	0.22 **	0.23 **	0.04
Fear of fat—Healthy weight control behaviors	0.16 **	0.13 **	−0.44
Fear of fat—Unhealthy weight control behaviors	0.33 **	0.53 **	3.21 **
Consciousness raising—Healthy weight control behaviors	0.34 **	0.32 **	−0.34
Counterconditioning—Healthy weight control behaviors	0.30 **	0.25 **	−0.71
Helping relationships—Healthy weight control behaviors	0.24 **	0.07	2.31 *
Reinforcement management—Healthy weight control behaviors	0.31 **	0.25 **	−0.73
Self-liberation—Healthy weight control behaviors	0.46 **	0.28 **	−2.64 **
Self-reevaluation—Healthy weight control behaviors	0.33 **	0.17 **	−2.25 *
Stimulus control—Healthy weight control behaviors	0.40 **	0.26 **	−1.96 *
Substance use—Healthy weight control behaviors	0.16 **	0.09 *	0.94
Consciousness raising—Unhealthy weight control behaviors	0.21 **	0.29 **	−1.06
Counterconditioning—Unhealthy weight control behaviors	0.25 **	0.35 **	−1.44
Helping relationships—Unhealthy weight control behaviors	−0.02	−0.12 **	1.33
Reinforcement management—Unhealthy weight control behaviors	0.19 **	0.07	1.68
Self-liberation—Unhealthy weight control behaviors	0.13 *	0.12 **	0.09
Self-reevaluation—Unhealthy weight control behaviors	0.27 **	0.38 **	−1.62
Stimulus control—Unhealthy weight control behaviors	0.19 **	0.23 **	−0.58
Substance use—Unhealthy weight control behaviors	0.36 **	0.49 **	−2.15 *

Note: ** *p* < 0.01, * *p* < 0.05.

**Table 4:** Results of values of correlation differences by country for the study variables.

Variables Correlated	Mexico (*n* = 722) Correlation	Spain (*n* = 116) Correlation	Fisher z Coefficient
Fear of fat—Consciousness raising	0.38 **	0.58 **	−2.66 *
Fear of fat—Counterconditioning	0.33 **	0.53 **	−3.78 **
Fear of fat—Helping relationships	−0.06	−0.05 *	0.10
Fear of fat—Reinforcement management	0.08 *	0.31 **	−3.69 **
Fear of fat—Self-liberation	0.20 **	0.43 **	−3.70 **
Fear of fat—Self-reevaluation	0.60 **	0.77 **	−3.61 **
Fear of fat—Stimulus control	0.36 **	0.43 **	−1.32
Fear of fat—Substance abuse	0.24 **	0.08	2.30 *
Fear of fat—Healthy weight control behaviors	0.12 **	0.30 **	−1.82
Fear of fat—Unhealthy weight control behaviors	0.48 **	0.51 **	−0.69
Consciousness raising—Healthy weight control behaviors	0.30 **	0.50 **	−3.50 **
Counterconditioning—Healthy weight control behaviors	0.24 **	0.39 **	−2.50 *
Helping relationships—Healthy weight control behaviors	0.14 **	0.06	1.13
Reinforcement management—Healthy weight control behaviors	0.27 **	0.32 **	−0.81
Self-liberation—Healthy weight control behaviors	0.32 **	0.46 **	−2.59 **
Self-reevaluation—Healthy weight control behaviors	0.21 **	0.38 **	−2.67 **
Stimulus control—Healthy weight control behaviors	0.29 **	0.46 **	−1.98 *
Substance use—Healthy weight control behaviors	0.14 **	−0.04	2.65 **
Consciousness raising—Unhealthy weight control behaviors	0.28 **	0.26 **	0.35
Counterconditioning—Unhealthy weight control behaviors	0.32 **	0.31 **	0.24
Helping relationships—Unhealthy weight control behaviors	−0.07	−0.23 **	2.39 *
Reinforcement management—Unhealthy weight control behaviors	0.10 **	0.16	−0.95
Self-liberation—Unhealthy weight control behaviors	0.12 **	0.17	−0.85
Self-reevaluation—Unhealthy weight control behaviors	0.36 **	0.38 **	−0.41
Stimulus control—Unhealthy weight control behaviors	0.24 **	0.21 *	0.39
Substance use—Unhealthy weight control behaviors	0.47 **	0.31 **	2.83 **

Note: ** *p* < 0.01, * *p* < 0.05.

**Table 5:** Distribution of the categorization of the sample in stages of change (*n* = 838).

Have You Tried to Lose/Control Your Weight?	*Stage*	f	%	Male %	Female %
Yes, I have done it for more than 6 months	*Maintenance*	*271*	32.3	24.3	36.1
Yes, I have done it for less than 6 months	*Action*	*342*	40.8	38.8	41.5
No, but I will try in the next 30 days	*Preparation*	*80*	9.4	13.7	7.6
No, but I will try in the next 6 months	*Contemplation*	*54*	6.4	8.6	5.5
No, and I do not intend to try in the next 6 months	*Precontemplation*	*91*	10.9	14.5	9.2
	Total	*838*	100		

Note: f = frequency; % = percentage.

**Table 6:** MANOVA testing differences between stages of change and processes of change.

Variables	*F*	df	*p*	Eta	Levene	*p*
Consciousness raising	42.85	4	0.01	0.17	5.51	0.01
Counterconditioning	22.67	4	0.01	0.09	4.65	0.01
Helping relationships	3.71	4	0.05	0.01	7.08	0.01
Reinforcement management	21.71	4	0.01	0.09	2.92	0.02
Self-liberation	48.37	4	0.01	0.18	7.24	0.01
Self-reevaluation	37.75	4	0.01	0.15	13.26	0.01
Stimulus control	28.69	4	0.01	0.12	2.40	0.01
Substance use	9.16	4	0.01	0.04	14.32	0.01

Note: df = degrees of freedom.

**Table 7:** Processes of change as mediators between fear of fat and healthy and unhealthy weight control behaviors.

Dependent Variables Predictors	B	SE	t	R^2^
Consciousness raising	0.43	0.03	12.55 **	0.18 **
Counterconditioning	0.46	0.04	11.04 **	0.12 **
Helping relationships	−0.08	0.05	−1.48	0.00
Reinforcement management	0.13	0.04	2.88 **	0.04 **
Self-liberation	0.34	0.04	7.09 **	0.08 **
Self-reevaluation	0.93	0.04	22.71 **	0.40 **
Stimulus control	0.46	0.04	11.16 **	0.15 **
Substance use	0.21	0.03	6.46 **	0.06 **
Fear of fat				
Healthy weight control behaviors				0.20 **
Consciousness raising	0.19	0.04	4.74 **	
Counterconditioning	0.06	0.03	1.74	
Helping relationships	−0.03	−0.08	0.02	
Reinforcement management	0.05	0.02	−1.11	
Self-liberation	0.15	0.03	4.51 **	
Self-reevaluation	−0.05	0.03	−1.47	
Stimulus control	0.11	0.03	2.70 *	
Substance use	−0.03	−0.04	−0.65	
Unhealthy weight control behaviors				0.40 **
Consciousness raising	0.00	0.02	0.01	
Counterconditioning	0.11	0.02	5.26 **	
Helping relationships	−0.05	0.01	−2.89 **	
Reinforcement management	−0.02	0.02	−0.74	
Self-liberation	−0.01	0.02	−0.62	
Self-reevaluation	0.04	0.02	1.61	
Stimulus control	−0.06	0.02	−2.36	
Substance use	0.31	0.02	12.46 **	

Note: B = unstandardized regression coefficient; SE = standard error; t = t-value; R^2^ = coefficient of determination. * *p* < 0.05; ** *p* < 0.01.

**Table 8:** Indirect effects of fear of fat on healthy and unhealthy weight control behaviors.

Indirect Effect [Mediator]	Indirect Effect	Bootstrap LL 95% CI	Bootstrap UL 95% CI
Healthy weight control behaviors			
[Consciousness raising]	0.08	0.047	0.123
[Counterconditioning]	0.03	−0.002	0.059
[Helping relationships]	0.00	−0.002	0.010
[Reinforcement management]	0.01	−0.003	0.021
[Self-liberation]	0.05	0.026	0.082
[Self-reevaluation]	−0.05	−0.117	0.016
[Stimulus control]	0.04	0.012	0.087
[Substance use]	−0.01	−0.025	0.012
Unhealthy weight control behaviors			
[Consciousness raising]	0.00	−0.021	0.022
[Counterconditioning]	0.05	0.031	0.074
[Helping relationships]	0.00	−0.001	0.011
[Reinforcement management]	−0.00	−0.009	0.003
[Self-liberation]	−0.00	−0.020	0.011
[Self-reevaluation]	0.03	−0.007	0.074
[Stimulus control]	−0.02	−0.049	−0.005
[Substance use]	0.07	0.038	0.099

Note: LL = lower limit, UL = upper limit, CI = confidence interval.

## Data Availability

All data used in this study are presented in the manuscript.
